# A new measurement for posterior tilt predicts reoperation in undisplaced femoral neck fractures: 113 consecutive patients treated by internal fixation and followed for 1 year

**DOI:** 10.3109/17453670902967281

**Published:** 2009-06-01

**Authors:** Henrik Palm, Kasper Gosvig, Michael Krasheninnikoff, Steffen Jacobsen, Peter Gebuhr

**Affiliations:** Department of Orthopaedic Surgery, Hvidovre University HospitalCopenhagenDenmark

## Abstract

**Background and purpose** Preoperative posterior tilt in undisplaced (Garden I–II) femoral neck fractures is thought to influence rates of reoperation. However, an exact method for its measurement has not yet been presented. We designed a new measurement for posterior tilt on preoperative lateral radiographs and investigated its association with later reoperation.

**Patients and methods** A consecutive series of 113 patients, ≥ 60 years of age with undisplaced (Garden I–II) femoral neck fractures treated with two parallel implants, was assessed regarding patient characteristics, radiographs, and rate of reoperation within the first year. In a subgroup of 50 randomly selected patients, reliability tests for measurement of posterior tilt were performed.

**Results** Intra-and interclass coefficients for the new measurement were ≥ 0.94. 23% (26/113) of patients were reoperated and increased posterior tilt was an accurate predictor of failure (p = 0.002). 14/25 of posteriorly tilted fractures ≥ 20° were reoperated, as compared to 12/88 of fractures with less tilt (p < 0.001). In multiple logistic regression analysis including sex, age, ASA score, cognitive function, new mobility score, time from admission to operation, surgeon's expertise, postoperative reduction, and implant positioning, a preoperative posterior tilt of ≥ 20° was the only significant predictor of reoperation (p < 0.001).

**Interpretation** The new measurement for posterior tilt appears to be reliable and able to predict reoperation in patients with undisplaced (Garden I–II) femoral neck fractures.

## Introduction

Femoral neck fractures (FNFs) are usually classified using Garden's classification into undisplaced (I–II) or displaced (III–IV) fractures, assessed from preoperative anterior-posterior (AP) radiographs (Garden 1961). Undisplaced (Garden I–II) fractures are usually treated with internal fixation (IF) using various parallel implants. A more refined decision-making strategy for these patients is needed, as overall reoperation rates are 8–20% (Alho et al. 1991, 1992, Parker et al. 2001, 2007, Conn and Parker 2004, Bjorgul and Reikeras 2007).

Few authors and classification systems have hypothesized that there is an influence of posterior angular displacement (tilt) on late surgical outcome in apparently undisplaced FNFs (Muller 1980, Alho et al. 1991, 1992, Orthopaedic Trauma Association Committee for Coding and Classification 1996, Conn and Parker 2004, Bjorgul and Reikeras 2007, Orthopaedic Trauma Association Classification, Database and Outcome Committee 2007). Alho et al. (1992) did not find a statistically significant effect of posterior tilt in 149 cases of FNF, but the study included both undisplaced and displaced fractures. In a retrospective study of 375 IF patients, Conn and Parker (2004) found that the presence of posterior tilt had a statistically significant, negative effect on nonunion rates, but not on the rate of subsequent avascular necrosis. None of the previous studies have, however, described an exact method for measurement of the posterior tilting.

The aims of our study were (1) to invent a new reliable measurement for posterior tilt, and (2) to investigate the influence of the measured posterior tilt on surgical outcome in undisplaced (Garden I–II) fractures after IF with two parallel implants.

## Patients and methods

113 consecutive patients aged 60 years or older (mean age 78 (60–99) years, 82 women) were admitted to our department between September 2002 and November 2006 with Garden I–II fractures (undisplaced inferior cortical buttress (Garden 1961)) treated in a fracture table by IF with 2 parallel implants: Olmed screws (Olmed Medical AB, Sweden) in 37 cases, and Hansson pins (Swemac Orthopaedics AB, Sweden) in 76 cases.

The patients followed the department's multimodal fast-track hip fracture program (Foss et al. 2005). They underwent daytime surgery using epidural anesthesia. Preoperatively, a single dose of 1.5 g cephalosporin was administered intravenously. Postoperatively, low-molecular-weight heparin was administered until full mobilization. Mobilization with full weight bearing was encouraged from the first day of surgery in a physiotherapy program with two daily sessions. Patients were scored according to the American Society of Anaesthesiologists Physical Grading Score (ASA 0–4) (American Society of Anaesthesiologists 1963), and Parker's New Mobility Score (NMS 0–9, where ≤ 5 designates inhibited functional level) (Parker and Palmer 1993). Patient's cognitive function was assessed with a Danish version of the abbreviated mental status test taken upon admission (Quereshi and Hodkinson 1974). The expertise of the surgeon was determined and scored as a junior registrar procedure or as senior surgeon procedure (Palm et al. 2007). Patient data were prospectively included in a database.

**Figure 1.**
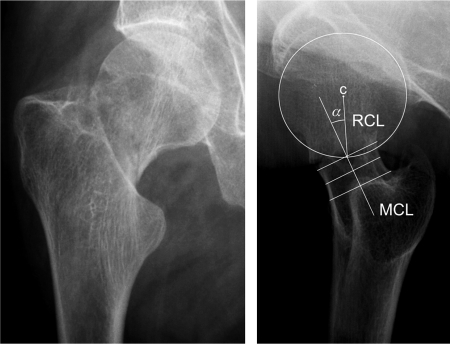
Preoperative anterior-posterior and lateral radiographs of a 60-year-old male patient who sustained a Garden I–II femoral neck fracture. The posterior tilt is measured as the angle (α) between the mid-collum line (MCL) and the radius collum line (RCL), which is drawn from the center (c) of the caput circle to the crossing of the caput circle and the mid-collum line.

Radiographs were stored in the Image Management and Applications-Radiology Information Service (IMPAX-RIS) system (Agfa, Köln, Germany) and digitally measured retrospectively. Posterior tilt was determined in preoperative lateral radiographs as the angle between (1) the mid-collum line (MCL) and (2) the radius collum line (RCL) (Figure 1). MCL was drawn through the middle of 3 perpendicular lines across the collum; with 1 line drawn at the narrowest part of the collum, and 2 parallel lines drawn subsequently 5 mm apart on each side. RCL was drawn from the center of the caput circle to the crossing of the caput circle and the mid-collum line.

All measurements were assessed by the same observer (HP). For reliability reasons, an intra-and interobserver study was performed by 2 of the authors (HP and KG, who was junior orthopedics resident) on 50 randomly selected lateral radiographs with independent assessment of posterior tilt twice, 2 weeks apart. At the time of assessment, the observers were blinded regarding postoperative radiographs and which patients later required a reoperation.

All fractures remained undisplaced in the first postoperative AP radiograph and fracture reduction was therefore assessed purely as postoperative posterior tilt in the first postoperative lateral radiograph. Implant positioning was assessed from AP and lateral radiographs as the minimal perpendicular distance (in mm) from the implants to the outer cortex contrast line of (1) the calcar, and (2) the posterior cortex, both on the femoral shaft side of the fracture.

Reoperations within 1 year were registered from patient records and cross-checked with the Copenhagen radiological database for admission due to complications to hip surgery in other departments. Only reoperations due to technical failures—fracture displacement, nonunion, avascular necrosis, subsequent fractures round the implant, or cutout of implant from the femoral head—were assessed as outcome parameter. All patients were scheduled for a follow-up visit including radiographs at 6 weeks postoperatively. If delayed but possible signs of healing were observed, several radiographs were later performed. All patients with radiographs showing technical failures were reoperated.

The study was part of the hip fracture project at Hvidovre University Hospital, Copenhagen, Denmark. It was approved by the Danish data protection agency and Copenhagen ethics committee. The latter concluded that the nature of the study was such that written consent from patients was not required.

### Statistics

The time between the beginning of our department's multi-modal fast-track hip fracture program (Foss et al. 2005) and the present study decided the number of patients included. Differences in perioperative parameters were analyzed using chi-square test for dichotomized values and Mann-Whitney test for continuous values. Survival between groups was analyzed using Kaplan-Mayer survival tables. Finally, demographic and clinical parameters that might hypothetically influence reoperation rate were entered into multivariate regression analyses. Intra-and interobserver variability were analyzed by intraclass coefficients. Level of significance was set at p < 0.05. All calculations were performed using the SPSS statistical software version 16.0.

## Results

31 of the 113 patients (27%) had reoperations within the following year. 3 of these patients had implants removed after successful healing due to skin problems and soft tissue pain, and 2 patients had to be reoperated due to superficial or deep infection. The remaining 26 patients (23%) had additional surgery performed due to technical failures (outcome parameter): 13 with fracture displacement and nonunion, 4 with nonunion in an undisplaced fracture position, 4 due to avascular necrosis, 3 due to subsequent fractures around the implants, and 2 due to cutout of one of the implants into the hip joint. No differences attributable to having sustained a Garden I or a Garden II fracture (4/16 vs. 22/95, p = 0.9) or selecting Olmed screws or Hansson pins (9/37 vs. 17/76, p = 0.8) were observed.

Assessment of posterior tilt was possible in all patients. The interclass coefficient (95% CI) for measurement of posterior tilt in the 50 randomly selected patients was 0.94 (0.91–0.97), with an intraclass coefficient varying from 0.95 (0.91–0.97) for KG to 0.97 (0.95–0.98) for HP.

**Figure 2.**
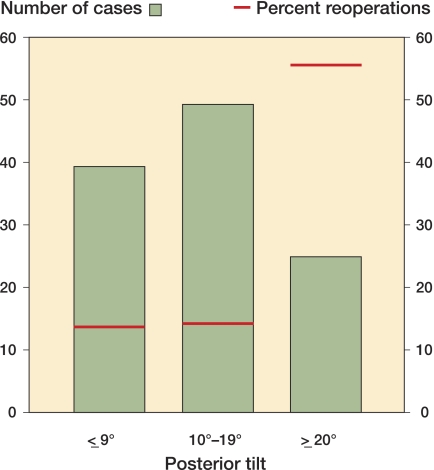
Distribution of posterior tilt and rates of reoperation in the 113 patients who were operated on with internal fixation for a Garden I–II femoral neck fracture. Numbers in parenthesis are (reoperated patients / patients) in the relevant group.

Figure 2 shows the distribution of posterior tilt and the rate of reoperation in relevant groups. Mean posterior tilt was 13° (range -8 to 47). 2 fractures angulated anteriorly and were assessed as a negative posterior tilt. Increased posterior tilt was found to be predictive of reoperation (p = 0.002). Based on highest sensitivity and specificity (ROC curve data not shown), posterior tilt was then dichotomized into < 20° or ≥ 20°. No statistically significant differences were found between these 2 groups in terms of gender, age, ASA score, new mobility score, time from admission to operation, surgeon's expertise, postoperative reduction, or implant positioning. 14/25 (0.6) of patients with posterior tilt of ≥ 20° were reoperated, as compared to 12/88 (0.1) of patients with tilt of < 20° (p < 0.001) (Table 1). 19% (21/113) of the patients died within the first postoperative year, with no difference in average number of days (95% CI) of survival within the study between the two above-mentioned groups of posterior tilt (321 (280–363) days vs. 322 (301–344) days, p = 0.7). In a logistic regression analysis combining sex, age, ASA score, cognitive function, new mobility score, time from admission to operation, surgeon's expertise, postoperative reduction, and implant positioning, a posterior tilt of ≥ 20° was the only significant predictor of reoperation (p < 0.001) (Table 2).

**Table 1.** Data for the 113 patients operated on with internal fixation for a Garden I–II femoral neck fracture

	Posterior tilt < 20 °	Posterior tilt ≥ 20 °	p-value
Number of patients **^a^**	88 (100)	25 (100)	
Female sex **^a^**	66 (75)	16 (64)	0.3
Age (years) **^b^**	80 (75–86)	74 (66–87)	0.09
Prefracture NMS **^c^** 0–5 **^a^**	40 (46)	10 (40)	0.6
ASA **^d^** score III–IV **^a^**	37 (42)	10 (40)	0.9
Low cognitive function **^a^**	27 (31)	4 (16)	0.1
> 1 day from admission to operation **^a^**	8 (9)	1 (4)	0.4
Senior surgeon procedure **^a^**	70 (80)	20 (80)	1
Postoperative posterior tilt (degrees) **^b^**	6 (0–10)	7 (2–14)	0.2
Implant distance to calcar (mm) **^b^**	6 (4–8)	6 (5–8)	0.3
Implant distance to posterior cortex (mm) **^b^**	5 (3–7)	4 (2–6)	0.4
Reoperation within 1 year **^a^**	12 (14)	14 (56)	< 0.001

^a^ Number of patients (percentage) and p-values determined with the chi-square test.

^b^ Median (interquartile range) and p-values derived with the Mann-Whitney test.

**^c^**NMS: new mobility score.

^d^ ASA: American Society of Anesthesiologists.

**Table 2.** Relationship between reoperation within 1 year postoperatively and patient characteristics for the 113 patients operated on with internal fixation for a Garden I–II femoral neck fracture

		Reoperation within 1 year postoperatively			
		Univariate analysis		Multivariate analysis	
		Odds ratio (95% CI)	p-value	Odds ratio (95% CI)	p-value
Female sex **^a^**	82 (73)	3.6 (1–13)	0.04	3.0 (0.7–13)	0.1
Age (years) **^b^**	79 (72–86)		0.9	1.0 (1.0–1.0)	0.4
Prefracture NMS **^c^** 0–5 **^a^**	50 (44)	0.5 (0.2–1.2)	0.1	0.5 (0.2–1.9)	0.3
ASA **^d^** score III–IV **^a^**	47 (42)	0.7 (0.3–1.7)	0.4	0.8 (0.3–2.5)	0.7
Low cognitive function **^a^**	31 (27)	0.4 (0.1–1.3)	0.1	0.9 (0.2–3.6)	0.8
> 1 day from admission to operation **^a^**	9 (8)	1.0 (0.2–4.9)	1	1.3 (0.2–8.7)	0.8
Senior surgeon procedure **^a^**	90 (80)	0.8 (0.3–2.3)	0.7	0.8 (0.2–2.7)	0.7
Postoperative posterior tilt (degrees) **^b^**	6 (0–11)		0.7	1.0 (0.9–1.1)	0.4
Implant distance to calcar (mm) **^b^**	6 (4–8)		0.2	0.9 (0.7–1.1)	0.2
Implant distance to posterior cortex (mm) **^b^**	5 (3–7)		0.3	1.0 (0.8–1.1)	0.6
Posterior tilt ≥ 20° **^a^**	25 (22)	8.0 (3–22)	< 0.001	13 (4–46)	< 0.001

**^a–d^**See Table 1

## Discussion

Femoral neck fractures are usually classified according to caphalad-caudal displacement on an AP radiograph of the pelvis. Here we present a new measurement for posterior angular displacement, which appears to be both reliable and an important predictor of subsequent reoperation due to technical failure of internal implants within 1 year. Although statistically significant as a continuous parameter, we have chosen to present our new measurement for posterior tilt as a dichotomized value, as the knowledge of “50% failure when preoperative posterior tilt exceeds 20 degrees” can be used in everyday clinical practice.

Our study could be questioned for grouping 2 types of IF implants together. However, we find this acceptable since in this study and in previous studies we have not found differences in rates of reoperation between the 2 types of IF (Parker et al. 2001, Mjorud et al. 2006).

The proximal femur and its accompanying vessels are naturally a complex 3-dimensional structure, and it is not surprising that surgical outcome after FNF is also influenced by fracture displacement along the lateral plane. It is, however, surprising that neither the positioning of the implants nor fracture reduction seemed to compensate for the damage caused by lateral displacement in the fracture. This might be explained by (1) irreversible damage of the vessels, resulting in avascular necrosis, and/or (2) a more unstable, geometrical fracture pattern that is at a higher risk of subsequent fracture displacement, resulting in pain and nonunion and the need for reoperation.

Garden introduced the alignment index for reducing angulation in both AP and lateral radiographs, but somehow this consideration was not included in his classification (Garden 1961). Muller's original AO classification suggested an influence of the posterior tilt (Muller 1980), which has been further developed in subsequent revisions of the AO/OTA fracture classification, but only in infrequently used subgroups (Orthopaedic Trauma Association Committee for Coding and Classification 1996, Orthopaedic Trauma Association Classification, Database and Outcome Committee 2007).

Alho et al. (1992) did not find an effect of posterior tilt using multivariate analysis on 149 FNF patients treated by IF. The reason for this could be the larger influence of cephaladcaudal displacement seen on AP radiographs, as patients with both Garden I–II and Garden III–IV fractures were included. Conn and Parker (2004) did, however, find that posterior tilt statistically significantly (albeit slightly so) influenced the rate of nonunion after Garden I–II fractures, but not the rate of avascular necrosis in a study of 375 IF patients. Recently, Bjorgul and Reikeras (2007) found a reoperation rate due to healing disturbance of only 9% for Garden I–II fractures operated on with 2 parallel screws when excluding patients with a posterior tilt of more than 30 degrees. The latter patients were grouped as moderately displaced fractures, for which internal fixation was doubted as being an adequate method.

Our findings confirm those of Conn and Parker (2004) and Bjorgul and Reikeras (2007). However, it is not clear how Alho and co-workers—to whom Bjorgul and Reikeras refer their measurements—actually measured the posterior tilt (Alho et al. 1991, 1992, Bjorgul and Reikeras 2007). Conn and Parker (2004) referred to the lateral Garden angle/alignment index that Garden (1961) originally determined by measuring the angulation between the trabeculae in the femoral head center and in the femoral neck. To our knowledge, the method has not been tested between different observers and in our opinion it is difficult and unreliable, as the quality of the lateral radiographs is often poor and the trabeculae invisible, especially in obese patients. As our method only uses the outer cortex contrast lines of the collum and caput, we could assess the posterior tilt in all our patients with an acceptable intraand inter-reader reliability. Also, the method is quite easy and could possibly be used in everyday clinical practice.

Initially, we tried to distinguish between displacement and angulation, but found it too unreliable. The posterior tilt measured according to our recommendation summarises both posterior angulation and displacement between the caput sphere and the collum cylinder, both of which may influence the stability of the fracture and the vessels passing the fracture area—and thus explain our findings.
